# Managing health care needs of the elderly through an elderly care manager: Thailand

**DOI:** 10.12688/f1000research.122555.1

**Published:** 2022-06-21

**Authors:** Chanuttha Ploylearmsang, Chutamat Somboon, Utoomporn Namdee, Saithip Suttiruksa

**Affiliations:** 1 International Primary Care Practice Research Unit, Faculty of Pharmacy, Mahasarakham University, Kantarawichai, Maha Sarakham, 44150, Thailand; 2 Faculty of Pharmacy, Mahasarakham University, Kantarawichai, Maha Sarakham, 44150, Thailand

**Keywords:** Elderly Care Manager, Community, Older people, Health needs, Quality of life

## Abstract

**Background**: This mixed method research aimed to investigate health needs of older people and the attributes of the Elderly Care Managers (ECMs), and to evaluate the outcomes of two ECMs coordination.

**Methods**: Three phases were: 1) a field survey of the health needs of 94 older persons; 2) group discussions between ten relevant persons involved in ECMs characteristics; 3) two ECMs coordinating with health-related agencies and measuring the outcomes of older people who needed assistance.

**Results**: 63.1% of the participants had difficulties with their health, 12.8% of them had no caregiver, 26.6% of them had >1 health condition. Group talks dealt with the characteristics, role and attributes of ECMs. The two-month of ECMs coordination with health-related agencies according to older adults’ needs, and made home visits encouraged the aged to acquire knowledge on their diseases.

**Conclusion**: ECMs’s coordination with health-related agencies could support the needs of those of advanced age.

## Introduction

An aging society is defined as one in which those who are 60 years of age or older make up more than 10% of the whole population of the country. In Thailand, the proportion of elderly persons in society has continuously increased as the birth rate continues to fall, and people live longer. By the year 2022, the number of Thai people who are 60 years of age and above will increase to 20 million, or 20% of the whole population ( The National Statistical Office of Thailand, 2019). This change in the population characteristics is predicted to result in a dependency ratio, with regard to the elderly, will rise from 47.4% in 2019 to 99.8% in 2037 ( Foundation of Thai gerontology research and development institute, 2020).

An elderly person faces both physical and mental changes. The immune system is weakened, resulting in generally more health problems than those in other age groups. It is also found that most aged individuals have chronic illnesses, especially dementia, which affect their daily lives and their quality of life ( Barbe *et al.*, 2018). As their ability to take care of themselves decreases, and as they are left alone (while the rest of their family members go out to work), people of advanced age often suffer from depression. Additionally, the environment that they live in is unsuitable to physical changes. experienced by elderly individuals.

A long-term care system is extremely necessary for an aging society. The World Health Organization ( WHO, 2020), has stipulated that long-term care is an indispensable part of the health system of society. There needs to be care activities by both formal caregivers, who are public officials, and medical practitioners in their areas, and informal caregivers, who are members of the family, friends and neighbors ( Laranjeira, 2020). Important factors for the success of the long-term care system are the existence of supporting policies and regulations, a good, systematic, structure of services, channels of access to services, funding from various sources, flexible services and the ability to provide services to the residents of the older, participation from the community and relevant networks ( Canadian Foundation for Healthcare Improvement, 2020). An Elderly Care Manager (ECM), at the level of the community, from the community itself, can play the role of a coordinator who addresses the various factors, and finds different ways to manage and solve different problems in response to the individual needs of the elderly in different cases. It may be said that the goals of care are to ensure that the basic factors in the daily lives of the older are meaningfully addressed, whether from the point of view of their expectations, or in terms of the existing standards of care ( Yodpet, 2009).

Thus, the researchers were interested in exploring the health problems of the aged in a community, and that through conducting group discussions, getting the opinions of the relevant persons who are responsible for the care of the elderly in the community, and to investigate the outcome through ECM coordination. The aim of this research was to find out the outcomes when informal care in the community is carried out by members of the community themselves.

## Methods

This is mixed method research, using both quantitative methods for exploring the difficulties faced by the elderly and outcomes due to the ECM, and qualitative methods, based on focus group discussion. The relevant persons involved in elderly care, at this particular time, carried out their activities in Kruewan community1, city municipality, Maha Sarakham Province. The data collection in the field was carried out over a period of six months. The study was separated into three phases, each with a specific objective. The three phases were: 1) to explore the difficulties faced by older people in the community using an interview form ( Ploylearmsang, 2022a) that the researchers had designed, and a trained team of researchers who used this form to collect the necessary information; 2) to conduct focus group discussions with the relevant persons involved in the system of long-term care for the elderly in the community. They consisted of representatives from the municipality, spokespersons of the community leaders, envoys of the older from the community, Village Health Volunteer (VHV), and health practitioners in the area; and 3) to study the two months short-term outcomes that were enhanced by two trained volunteer ECMs who coordinated with health-related agencies to provide contributions or interventions according to the needs of elderly, made at least two home visits over a period of two months, and informed the elderly about the diseases and some supportive help for improving quality of life of the elderly.

### Population and samples

The three phases of the research had three different population and sample groups, as follows:

1) The population in the first stage consisted of the elderly persons in Kruewan1 community. The subjects, consisting of both males and females, were ≥60 years on the day of the survey. They were also residents of the Kruewan1 community throughout the three months period, and were willing to provide the data. The results were calculated based on the size of the sample group for the survey, p=ratio of aging patients with chronic illnesses–53% ( Gray, Hahn, Thongcharoenchupong, 2016) applying the Cochran formula (1953) as follows:
$$n=\frac{\times \left(1-\right)\times^{2}\times \left(1-\right)\times^{2}}{2\;2}=\frac{0.53\times \left(1-0.53\right)\times 1.96^{2}}{0.1^{2}}\approx \frac{0.53\times \left(1-0.53\right)\times 1.96^{2}}{∼0.1^{2}}\approx 96\;\text{individuals}$$
  n=the minimum number of sample groups needed
  Z _α/2_=constant under α error where α=0.05 then Z _α/2_=1.96
  e=acceptable error, equivalent to 10% or 0.1
  The research team conducted an interview of 45-60 minutes’ duration for each elderly person.
2) The population in the second phase consisted of relevant persons involved in the health care of the elderly in Kruewan community1. Those who were able to give evidence, and willing to participate in group discussions, contributed to the investigation and ECM training. There were ten individuals in total, consisting of one community leader, one representative of the elderly, three VHV, one municipality official in charge of the maintenance and improvement of housing conditions, and four health personnel who had worked in the research area: a doctor, a nurse, a pharmacist, and a physiotherapist.
3) The population in the third phase consisted of the elderly who participated in phase 1. Samples consisted of elderly persons who had made a request for healthcare and were willing to accept assistance from ECMs. The sample size was calculated using the effect size of paired data on quality of life.

n = [(Z _α/2_ + Z _β_)σ/Δ] ^2^ when Z _α/2_ = 1.96, Z _β_ = 0.84, and Δ of EQ 5D = 0.15 ( Comans *et al.*, 2013), n=13.9 or 14. Fourteen elderly persons that fitted the inclusion criteria were recruited. The exclusion criteria were: 1) patients that ECMs were unable to access or collect the data, because they had moved out of the area or passed away during the data collection period; 2) those that requested for discontinuation.

### Research instruments

1. An interview form and a consent form were used for collecting information regarding issues faced by the elderly in the community.
2. The questions were framed by the investigators for the group discussion. The subject matter included their view on ECM, the characteristics and roles of the ECM. The questions were checked for content validity by an expert on development of survey instruments and a local authority on health care for the elderly. The interview form was tested for reliability on ten elderly persons in other localities.

### Research process

The 10 steps for the research were: 1) submission of a human research ethics form to the Ethical Committee of xxxxx University with the ethical approval number xxxxx submission of a letter of request for assistance to Burapha primary care unit (PCU) in order to collect research data from populations in Kruewan 1 community; 3) submission of a request for permission to access the study area to the Maha Sarakham city municipality; 4) conducting a field survey of older people in order to find out about their situation and any problems affecting their health; 5) collection of data by tape recorded interviews, analyzing the results, and categorizing the problems and the health needs of people of the elderly; 6) leading a group discussion in order to exchange information on the conditions and difficulties faced by the elderly in the community, and recording of opinions held by members of the community about the characteristics, roles and responsibilities of the ECMs; 7) gathering of evidence from group dialogues, identification of the subject matters discussed, and analysis of the resultant evidence; 8) categorization of the difficulties faced by the elderly based on the information obtained from the first phase of the survey, and training ECMs to coordinate with specific personnel or agencies who have the ability to resolve individual problems, together with suggestions and solutions to be applied by the relevant agency; 9) collecting paired data for the supportive effects of ECMs’ coordination on short-term outcomes among the elderly; 10) summarizing data and providing feedback to the community.

### Ethical approval

Approval IRB protocol/human subjects approval numbers: Faculty of Pharmacy Mahasarakham University Ethics Board (REB: PD 014/2014).

### Statistical analysis

The relevant general and health information of the participants during the first stage were described by descriptive statistics such as frequency, percent, average, and standard deviation. The paired data of pre-post supportive effects including score of disease knowledge (score 0-5) and quality of life (score 0-1) were analyzed by using Wilcoxon signed ranks tests.

## Results

### Problems and the needs of the older persons in the community

According to the information from Burapha Primary Care Unit (PCU), the population of Kruewan1 community consisted of 1,006 individuals. There were 149 elderly persons (9.34%) and 94 elderly persons were willing to participate in this survey, a response rate of 63.1%. There were 55 individuals whose information could not be obtained as they had moved, were not home at the time of the survey, or had passed away. Most of the older people in the Kruewan1 community were female (74.47%). As shown in Table 1, 53 individuals (56.38%) were 60-70 years old. 51 older persons (54.26%) were married and were together. In this community, there were forty aged persons (42.55%) who were widowed. The education level of most participants was at primary school level (93.62%). There were seventy-nine elderly persons (84.04%) who were no longer in any occupation. Eighty-two of the elderly had regular caregivers (87.23%), but as many as twelve elderly persons (12.77%) had no regular caregivers.

**Table 1.** General information about elderly persons in Kruewan 1 community (n=94).

General information	Numbers (persons)	Percentage
1) Gender		
Male	24	25.53
Female	70	74.47
2) Age (average age 70.26 ± 6.91 years old)		
60-70 years old	49	52.13
71-80 years old	38	40.42
≥ 81 years old	7	7.45
3) Marital status of older persons		
Single	1	1.06
Married and together	51	54.26
Divorced/separated/Widowed	42	44.68
4) Highest level of education		
Primary	88	93.62
Secondary/vocational/certificate	3	3.19
Bachelor degree	3	3.19
5) Main occupation		
No occupation	79	84.04
In occupation	15	15.96
−Rice farming	4	4.26
−Private business	7	7.44
−General employment	4	4.26
6) Has a regular caregiver		
No caregiver	12	12.77
Has regular caregiver	82	87.23
child	63	67.02
husband/wife	16	17.02
relative	3	3.19
7) Underlying medical conditions		
No underlying medical condition	37	39.36
With underlying medical condition	57	60.64
1 medical condition	32	34.04
≥ 2 medical conditions	25	26.60
8) Types of underlying medical conditions		
High blood pressure	36	38.30
Diabetes	31	32.88
Hyperlipidemia	18	19.15
Gout, Rheumatoid Arthritis	6	6.38
Heart disease	5	5.32
Other medical conditions	17	14.89
9) 5 most common health problems among the older		
- Aches in the joints, knees and body	30	31.91
- Problems with mobility and movement, dizziness	9	9.57
- Problems with eyesight and visual acuity	4	4.26
- Problems with oral and dental health	3	3.13
- Problems with excretory system (constipation)	1	1.06

The status of the elderly in Kruewan1 community is shown in Table 2, and fifty-seven (60.64%) had underlying medical conditions. The three most common underlying diseases were high blood pressure (38.30%), diabetes (32.98%), and hyperlipidemia (19.15%). Other medical conditions which were found were: emphysema, migraine, hemorrhoids, Parkinson’s disease and partial paralysis. Five individuals were discovered to have them. Three were identified as having Alzheimer’s disease.

**Table 2.** The relevant persons’ opinions about ECM in the community.

Characteristics	The relevant persons’ opinions about ECM in the community (n=10)
1. General characteristics of ECM	“… it has to be someone from the community so that they would know about the community itself. Outsiders have no idea who has what. It doesn’t matter how old they are. They just have to be someone from the community and be capable. Most importantly, they need to be publicly minded …” Male 1 “… they should be healthy and strong. They should be mentally strong too. We need someone who puts their heart into this and can travel around. They shouldn’t have a lot of burdens in their lives that would affect their work ….” Female 2 “… they should also be responsible … they need to be able to travel and own a vehicle …” Female 4 “… being publicly minded is very important. They also need to be responsible and flexible too …” Female 5 “… They should love what they do … I want everyone to be involved. Someone who puts their heart into their work, who has the time to coordinate and liaise, someone who is publicly minded. We need them to be willing – not forced into this …” Male 9 “… well, I think they need to understand the older well, don’t they? They need to understand the older but I don’t think it has something to do with their age, is it?” Female 10
2. Capacities, abilities, knowledge and basic skills of ECM	“… they should be literate and keep up with the world. They need to have some knowledge and understanding of health care …” Female 2 “… they should have all-round knowledge of the communities regarding what’s happening around there and what the needs of the elderly are. They need to be skilled in using social media too …” Female 8 “… they need to understand the context of the community, know the VHV of a particular zone. They should know who or which agency to contact for what matter. Female 6 “… they should know who to contact. They don’t really need to do it themselves but they should know the chairperson of the elderly group in the community. They should know different groups of the elderly, the temples, the homes and the schools. They should have planning skills – planning for their work and putting that into practice …” Male 7 “… communication skills – they need to be able to communicate with any person from the community and outside of the community, or external agencies. So, they need to know the VHV, the chairperson of the community, and the care workers …” Female 10
3. Roles and responsibilities of ECM	“… they need to survey the needs of the elderly first before they do anything for the elderly. So that they know who needs what …” Female 2 “… they need to find the elderly first. They need to know where the elderly persons are, and where they live. Normally, the VHV would carry out a survey for this kind of information in any case. So, a Manager needs to coordinate with a VHV. Once they coordinate with the VHV, they would know the information …” Female 8 “… well, they need to be able to coordinate with all points but not necessarily do it themselves. Anyway, once they have done their work, they need to find a conclusion to their work. They need to be able to tell the elderly in our community how things are. If they hear something from this house and that house, they need to be able to have a chat and pass on the info …” Female 10

**Health needs of the older people in the community**

From the interview about the need for health services among the aged in the community, we concluded that there are seven main needs, as follows: (1) income-generating occupations, (2) commodities for daily living, such as blankets, (3) monetary support, (4) basic knowledge on diseases and medication, (5) equipment to increase their quality of life, such as canes, walkers, and wheelchairs, (6) social activities such as aerobic exercise groups, (7) advice and suggestions to manage their mental health and depression after the loss of loved ones.

### Group discussion results

Various results ( Ploylearmsang *et al.*, 2022) were derived from the group discussions conducted among ten relevant persons who were involved in health care for the elderly (see Table 3). They consisted of five members of the community who were involved with the elderly, and five personnel from the public sector who were health care providers for elderly persons. The group discussion was held for them to give their opinions on the health problems faced by the elderly, and the characteristics, qualifications and work methods of the ECM. The opinions about the ECM in the community were shown in Table 2.

**Table 3.** Elderly needs, ECMs coordination for the elderly needs and their outputs.

Elderly persons characteristics	Elderly persons’ needs	ECMs coordination	Outputs
1. Female, 73 years old, with self-movement problem, needs care-giver to look after her for whole day	1. Walker 2. Training how to use a walker and move to the restroom by herself.	1. Coordinate with the hospital officer and a physical therapist 2. Make a first home visit with the physical therapist for patient assessment and a second visit for training how to use walker properly 3. Consult the municipal officer about the possibility of adjusting house if this is needed 4. Monitor and report the result of use of walker to the physical therapist	1. Getting a walker 2. Be able to move to the toilet by herself, and be able to stay at home alone with no caregiver.
2. Female, 78 years old, with anti-diabetic medication no adherence (her understanding of it and concern about the side-effects of the use of the medication in the long run)	1. Drug information 2. Drug adherence improvement	1. Coordinate with a community pharmacist (University pharmacy) 2. Make a home visit with the pharmacist for knowledge and drug adherence assessment and intervention 3. Make a second home visit with the pharmacist for drug monitoring	1. Understanding of disease and drug Information 2. Better drug adherence
3. Female, 64 years old, with ≥ 4 items of medication or polypharmacy	1. Drug information 2. Drug adherence improvement	1. Coordinate with a community pharmacist (University pharmacy) 2. Make a home visit with the pharmacist for drug adherence assessment and polypharmacy intervention 3. Make a second home visit with the pharmacist for drug monitoring	1. Drug information and understanding the importance of drug adherence 2. Improved some drug adherence
4. Male, 77 years old, with hypertension and afraid of having diabetes as other family members have it	1. Disease information (Hypertension and Diabetes) 2. Improvement on anti- hypertensive drug adherence	1. Coordinate with a community pharmacist (University pharmacy) 2. Make a home visit with the pharmacist for disease knowledge and drug adherence assessment and intervention 3. Make a second home visit with the pharmacist for giving disease information and drug monitoring	1. Disease information and self-monitoring for DM
5. Female, 73 years old, with hypertension and diabetes, lives alone, no care-giver, often forgets to take medicines	A reminder for drug taking	1. Coordinate with a community pharmacist (University pharmacy) 2. Make a home visit with the pharmacist for drug adherence assessment 3. Make a second home visit with the pharmacist for giving a reminder pill box and self-drug monitoring	1. A reminder pill box 2. Better drug adherence
6. Male, 79 years old, he was unable to remember his medication, lives with his wife who is his caregiver. Both of them were old	Two reminders big and different colored pill boxes (one per person)	1. Coordinate with a community pharmacist (University pharmacy) 2. Make a home visit with the pharmacist for drug adherence assessment and care-giver training 3. Make a second home visit with the pharmacist for giving reminder pill boxes and self-drug monitoring	1. Reminder pill boxes 2. Better drug Storage, separately for each person
7. Female, 80 years old, who needs to quit smoking after knowing her sister got sick from severe lung disease	Quit smoking	1. Coordinate with a community pharmacist (University pharmacy-Smoking cessation service) 2. Make a home visit with the pharmacist for smoking cessation intervention (pharmacist monitors via phone call for three months)	Receiving smoking cessation service (having herbal lozenges)
8. Female, 76 years old, with DM and hypertension	Physical exercise that is suitable for her physical body	1. Coordinate with a health volunteer in the community to make a group for aerobic dancing exercise 2. Inform the members in the aging club to join the activities	Having exercise thrice a week
9. Female, 76 years old, with chronic stress from the suffering of a couple passing away	Psychological consult	1. Coordinate with a primary care unit (PCU) officer for depression screening and give a primary consult Then PCU officer consult psychiatric doctor and give the older person some information 2. Coordinate with her family to know her situation 3. Monitor her symptoms	Getting Screening and primary consult from PCU officer

### Effects of ECMs coordination with health-related agencies on outcomes for the elderly

Data was obtained, over a two-month period, from fourteen elderly persons who were willing to participate in this research, during which two trained volunteer ECMs coordinated with health-related agencies which could provide contributions or interventions according to the needs of the elderly. The ECMs made home visits during the two months’ period. Five participants were missing during follow up, two elderly persons were reported as dead, and three had moved to their children’s homes in other areas and thus the response rate was 64.3%. Table 4 shows the activities that ECMs performed for the elderly in their community, and Table 4 shows outcomes of these activities among the elderly after the two-month period. The trend in terms of increased quality of life (EQ-5D-5L) among the elderly from pretest to posttest was 0.80±0.16, 0.82±0.15 (p=0.059), while that for knowledge about diabetes and hypertension increased significantly from 4.11±0.60 to 4.56±0.53 (p=0.046) and 4.11±0.60 to 4.89±0.33 (p=0.008), respectively.

**Table 4.** The supportive effects of ECMs coordination with health-related agencies on older people’s health outcomes.

Aging’s outcomes	Mean±SD (n=9)		p-value ^a^
	Pre-test	Post-test	
Diabetes knowledge (Score=0-5)	4.11±0.60	4.56±0.53	0.046 *
Hypertension knowledge (Score=0-5)	4.11±0.60	4.89±0.33	0.008 *
Quality of life (EQ-5D-5L) (Score=0-1)	0.80±0.16	0.82±0.15	0.059

^a^ Wilcoxon signed ranks test.

* statistical significance at p<0.05.

## Discussion

A study of the development of systems for long-term care service in the community, found that 67.6% of the elderly were female ( Loskultong & Sritanyarat, 2012). Research investigating health problems, issues with the use of medications, and the behaviors of the elderly in the community of Phramongkutklao Hospital, with regard to the use of medications, also found that most of the residents of advanced age were female ( Naiyapatana, 2010). The results of the current study are consistent with the two studies mentioned i.e. elderly female would be a group of concern in the future. It was found that the health problems of the elderly were chronic diseases including hypertension, diabetes, hyperlipidemia, gout and rheumatoid arthritis, heart disease, and asthma. Some of the elderly had more than one underlying medical condition. This is consistent with the findings of the survey on the six most common chronic illnesses suffered by people of advanced years in 2019, which was conducted by the National Statistical Office. It is also consistent with the study from home visits and the management of left-over medications in elderly persons with chronic illnesses. It was found that the five most common chronic illnesses, with the left-over medications, among the aged were high blood pressure, diabetes, stroke, hyperlipidemia, and cardiovascular diseases, ( Chantra & Moungkan, 2020) Studies carried out on the development of systems for long-term care services for the elderly in the community found that the most common morbidity suffered by the elderly was high blood pressure ( Loskultong & Sritanyarat, 2012; Naiyapatana, 2010). Additionally, the results of this study demonstrated that most of the elderly had more than one underlying condition, and long-term medications use was important.

Elderly persons, living without caregivers was one of main factors for the aggravation of health problems amongst elderly in Kruewan1 community. Most of their children worked in different areas, and thus the elderly had to live alone, without a caregiver. Physical changes experienced by advanced age also affect their health. The survey found problems relating to aches and pains in joints, knees and body. Additionally, there were problems with balance and dizziness, eyesight and visual acuity, oral and dental health, and the excretory system and this is similar to the findings of a study in Thailand ( Jarutach, 2007). A large number of the elderly have orthopedic illnesses, and illnesses linked to joints/muscles/ligaments, and most elderly persons have problems with visual acuity. However, in terms of mental health, most of the elderly people in this study did not suffer from depression and this is consistent with the situation in Thailand as a whole where it was found that Thai elderly persons had average mental health scores of 32.3 out of 45 points – which was within the normal level of the mental health ( Yiengprugsawan *et al.*, 2012).

Environmental factors may also play a role in the health problems of the elderly. This is because ideal environments for the elderly vary according to their individual limitations. If an environment allows an elderly person to help themselves, the consequence may be positive effects on such a person’s mental health. They can take pride in being able to fend for themselves, and do not feel that they are burdening their children. Research has been shown that elderly persons with mobility problems and whose difficulties have been managed, can move around more comfortably, and are able to perform more movements by themselves ( Tongsiri *et al.*, 2017). Similarly, it has been found that elderly persons living in urban areas need to use equipment, which are safe and not complicated to use, to assist with their mobility ( Lertpradit & Jarutach, 2020).

Teaching the elderly to have basic knowledge about illnesses and medications is important. Awareness about such issues needs to be created among them and their caregivers in order to prevent health problems that may result from a lack of such information. A study on the management of left-over medications among the elderly with chronic illnesses, found that the elderly needed knowledge with regards to use of medications, and the methods to use them appropriately. Most of the elderly tend to forget to take their medications or to take them at the appropriate time ( Chantra & Moungkan, 2020). Thus, in this study, the elderly indicated their need for this information, and their caregivers also were of the opinion that they needed to know more in order to provide good support to the elderly person in the family. The results of our research also showed that after such educational activities the knowledge scores in this area had increased among the elderly.

There should be a system of management with continuity for the health problems of the elderly, especially for their long-term health care. Investigations into the development of systems for long-term older care services in the community found a lack of policy for long-term health care for the elderly ( Loskultong & Sritanyarat, 2012). Thus, in this research, an attempt was made to introduce the working methods, roles and responsibilities of ECMs in the community, and to indicate how their work should be carried out. As Somkamlang & Kitreerawutiwong stated, the main roles and responsibilities of the ECM can be divided into two categories, according to whether they are care providers or care managers. In the system employed in Japan, there is an arrangement for elderly care service in the form of care management, with care managers as the persons who individually organize the format of care for older persons ( Trakoolngamden *et al.*, 2018). The voluntary elderly care managers have to be trained and certified by the state ( Kelsey & Laditka, 2009). In Thailand, the Ministry of Social Development and Human Security (2014) stated that in order to substantially and systematically help with the daily lives of the elderly, and to understand them, voluntary care managers must be able to analyze the situation, the problems and the feelings of the elderly, and the relevant target groups, as well as conduct home visits, so as to follow up on relevant facts and information, in order to help with the planning of care for the elderly.

In terms of the outcomes of elderly by ECMs, in this present study found that the 2-month community supportive intervention for Thai elderly by ECMs has significantly effect on patients’ knowledge score but there was no significant improvement in elderly’s quality of life by EQ 5D, even an increasing trend of elderly quality of life was found. Whereas a study (Rachasrimuang, 2018) revealed that the 18-week home visit intervention by youth volunteers for the elderly in the Thai rural community showed the significant improvement in EQ 5D (p<0.001). Another study in Australia also found similar result that the discharge program for elderly had positive effect on the six-month quality of life from EQ 5D 0.75±0.16 to 0.84±0.25 within 6 months ( Comans *et al.*, 2013). Comparing with two references, the improvement of quality of life needs the suitable duration for implementing the intervention for the elderly.

## Conclusion

Problems affecting the health of the elderly in Kruewan1 community are underlying medical conditions, aches and pains, mobility problems, problems with eyesight and visual acuity, problems with oral and dental health, and not being able to be self-dependent. The needs of the elderly in the community are related to other problems which were identified such as the need to have an income-generating occupation, the need for essential commodities, financial support, knowledge about illnesses they suffered and the medications, equipment for mobility and for exercise groups, and to have consultation regarding mental health issues arising from depression due to the loss of loved ones.

From the views which were expressed about ECMs by those involved in healthcare for the elderly in the community, members of the community, public sector personnel, and health professionals, it may be concluded that an ECM needed to be someone from the community who is healthy and responsible, who puts their heart into the work, is service-minded, is willing to take on their responsibilities, fully dedicates their time to their work, is flexible, able to travel in order to coordinate their work, understands the elderly, and can work alone or in a team. While the level of education attained by such individuals is not an issue, this person needs to be literate and have a basic knowledge of health care. They also need to know the situation and the needs of the elderly in the community, and the pertinent channels of coordination or communication with relevant agencies with regard to health care for the elderly. They need to have communication skills, the capability to plan well, and get along easily with those in their areas, or from relevant teams. They are required to use appropriate communication channels in order to coordinate with others both within and outside of the community. Additionally, they need to use the appropriate information technology and computing systems. Their main responsibilities are to coordinate with relevant agencies in order to be able to respond to the basic needs of the elderly in the community.

### Limitations of this research

The information on the health of elderly persons who are on the public sector database is not up to date. Some of the elderly no longer live in the community but their names are still on the database. This caused problems in the collection of complete information in the survey of the elderly persons’ health problems. Additionally, the elderly person’s ability to recall is decreased and their recollection may be significantly limited on such matters as their medication use. Interviews, therefore, had to be conducted in the presence of others as well, such as the caregivers and the families. ECMs coordination with health-related agencies, which can provide contribution or intervention according to health needs of the elderly, provides a connection between the community and health-related agencies, and can be said to be one supporting components in terms of improving the situation of people of the elderly. However, it may not show up in terms of direct effects on such outcomes. There are other significant influencing factors, such as interventions by health-related agencies, the compliance displayed by the elderly themselves to instructions that are prescribed to them and the level of support that they receive from their families, all play a role.

### What this paper adds

• The connection between health services and the elderly in the community is the crucial elements of the long-term care. Elderly Care Manager (ECM), a voluntary coordinator in the community, is a key person for making the smooth connection.
• The opinions of the relevant persons, health-related officers and lay persons, about the ECMs, as well as the working process to recruit ECMs were created.
• The supportive interventions coordinated by ECMs has significantly affected on elderly’s health needs.

### Applications of study findings

• Elderly participation in their own community to co-create, develop, and implement the health-related project with the local authorities, is significant strategy for enhancing both elderly empowerment and community engagement. It could be generalized in other aging community.
• ECMs, recruited by community will be accepted and allowed to coordinate with elderly for improving their quality of life. They will be able to provide continuous help, manage problems, and respond to the elderly’s health needs.
• Elderly home visit is an encouraging process to learn and understand their health needs. It could be applied for elderly in other area.

## Data availability

### Underlying data

Figshare: Managing Health Care Needs of the Elderly through an Elderly Care Manager: Thailand, https://doi.org/10.6084/m9.figshare.19961522.v2 ( Ploylearmsang *et al.*, 2022).

Figshare: Data coding, https://doi.org/10.6084/m9.figshare.20010527.v1 ( Ploylearmsang, 2022b).

### Extended data

Figshare: Questionnaire and Questions for group discussion, https://doi.org/10.6084/m9.figshare.20010536.v1 ( Ploylearmsang, 2022a).

Data are available under the terms of the Creative Commons Attribution 4.0 International license (CC-BY 4.0).

## References

[ref1] Barbe C Jolly D Morrone I : Factors associated with quality of life in patients with Alzheimer’s disease. *BMC Geriatr.* 2018;18(159):159–159. 10.1186/s12877-018-0855-7 29986669 PMC6038200

[ref2] Canadian Foundation for Healthcare Improvement: *Long-term success and Sustainability of Healthcare Improvement Guide.* 2020, April 1. Reference Source

[ref3] Chantra C Moungkan J : Cause of Leftover Drugs in Elderly patients with Chronic disease at Ruamjai Community health care Wangthong Phitsanulok Province. *Proceedings in the 11th Hatyai National and International Conference.* 2020;1957–1967.

[ref4] Comans TA Peel NM Gray LC : Quality of life of older frail persons receiving a post-discharge program. *Health Qual. Life Outcomes.* 2013;11:58. 10.1186/1477-7525-11-58 23587460 PMC3637078

[ref5] Foundation of Thai gerontology research and development institute (TGRI): *Situation of the Thai elderly 2019.* Nakhon Pathom: Printory Co. Ltd;2020.

[ref6] Gray RS Hahn L Thapsuwan S : Strength and stress: Positive and negative impacts on caregivers for older adults in Thailand. *Australas. J. Ageing.* 2016;35(2):E7–E12. 10.1111/ajag.12266 PMC5069609 26969906

[ref7] Hirakawa Y : Care manager as a medical information source for elderly people. *Med. Res. Arch.* 2016;4(5):1–12.

[ref8] Lertpradit N Jarutach T : Housing Conditions and Improvement Guidelines for the Elderly Living in Urban Areas. *Nakhara: Journal of Environmental Design and Planning.* 2020;18(1):117–138.

[ref9] Jarutach T : The Minimum Standard of Environment and housing for Thai Elderly. *Proceedings of The Eighth Pan-Pacific Conference on Occupational Ergonomics (PPCOE2007).* 2007. Reference Source.

[ref10] Kittirattanapaiboon P Tanaree A : Trend and associated factors of mental health in Thailand: a national survey 2018. *J. Ment. Health Thai.* 2020;28(2):121–135.

[ref11] Laranjeira C : Quality of life of informal caregivers for elderly people with dementia: A comparative study. *Alzheimers Dement.* 2020;16(Suppl. 7):e047273. 10.1002/alz.047273

[ref12] Naiyapatana W : Health Problems, Medicine-Used Problems, and Medicine-Used Behaviors among Elderly in the Community of Phramongkutklao Hospital Personnel’s Residence. *J. Nurs. Educ.* 2010;3(1):2–14.

[ref13] Loskultong P Sritanyarat W : Development of Long-Term Care Service System for Community Older Persons in the Context of a Secondary Care Hospital. *Graduate Research Conference (GRC) 2012. Khon Kean University.* 2012. Reference Source

[ref14] National Statistical Office: Size and structure of the population by age and sex. *Statistical Section.* 2019, October 1. Reference Source

[ref15] Rachasrimuang S Kuhirunyaratn P Bumrerraj S : Effectiveness of a Home Visit Programme by Youth Volunteers on Health-Related Quality of Life and Depression among Elderly Persons: Results from a Cluster Randomized Controlled Trial in Rural Thailand. *J. Med. Assoc. Thail.* 2018;101(5):189–195.

[ref16] Somkamlang S Kitreerawutiwong N : Long Term Care Manager in District Health System. *J. Nurs. Health Sci.* 2019;12(4):1–8.

[ref17] Sub-committee for the development of standards for elderly integrated care: *Manual of Voluntary Elderly Care Manager.* Thailand: Department of Older Persons, Ministry of Social Development and Human Security;2014. Reference Source

[ref18] Kelsey SG Laditka SB : Evaluating the Roles of Professional Geriatric Care Managers in Maintaining the Quality of Life for Older Americans. *J. Gerontol. Soc. Work.* 2009;52(3):261–276. 10.1080/01634370802609171 19308831

[ref19] Ploylearmsang C Somboon C Namdee U : Managing Health Care Needs of the Elderly through an Elderly Care Manager: Thailand. figshare. [Dataset]. 2022. 10.6084/m9.figshare.19961522.v2 PMC11401987 39281328

[ref20] Ploylearmsang C : Questionnaire and Questions for group discussion. figshare. [Extended Dataset]. 2022a. 10.6084/m9.figshare.20010536.v1

[ref21] Ploylearmsang C : Data Coding. figshare. [Dataset]. 2022b. 10.6084/m9.figshare.20010527.v1

[ref22] Takahashi T Kaihara S : Introduction to the Status -Function-Care (SFC) Method. *Journal of Japan Hospital Management Association.* 1992;29:247.

[ref23] Tongsiri S Ploylearmsang C Hawsutisima K : Modifying homes for persons with physical disabilities in Thailand. *Bull. World Health Organ.* 2017;95(2):140–145. 10.2471/BLT.16.178434 28250515 PMC5327935

[ref24] Trakoolngamden B Mongkolsinh V Srangthaisong D : The Evaluation of Care Manager Course on Elderly in Bangkok Metropolis of Kuakarun Faculty of Nursing, Navamindradhiraj University. *Kuakarun J. Nurs.* 2018;25(2):210–228.

[ref25] World Health Organization: *Long-term-care systems.* 2020, November 1. Reference Source

[ref26] Yiengprugsawan V Somboonsook B Seubsman S : Happiness, Mental Health, and Socio-Demographic Associations Among a National Cohort of Thai Adults. *J. Happiness Stud.* 2012;13(6):1019–1029. 10.1007/s10902-011-9304-4 23304071 PMC3538273

[ref27] Yodpet S : *Integrations of Long-term Care System for the Elderly in Thailand.* 1st ed Bangkok: J Print 2 Publishing House;2009.

