# Supplementary material for: Managing health care needs of the elderly through an elderly care manager: Thailand
Source: F1000Res. 2022 Jun 21;11:680. [Version 1] doi: 10.12688/f1000research.122555.1 (PMC11401987; doi:10.12688/f1000research.122555.1)
Supplement: Peer review history

# Reviewer report: Reviewer response for version 1

**DOI:** 10.5256/f1000research.134559.r184484

**Published:** 2024-09-14

**Authors:** Anne Wissendorff Ekdahl

**Affiliations:** 1 Lund University, Lund, Sweden

This is an interesting article about using ECMs to care for the growing older population in Thailand, of which more older people live alone.

 I don't understand the "xxx" in the research process, as the ethical approval number is later exposed.

 The findings of health needs seem plausible and relevant. I my opinion, the article could be indexed to support the growing health needs of the Thai population.

Is the work clearly and accurately presented and does it cite the current literature?

Yes

If applicable, is the statistical analysis and its interpretation appropriate?

I cannot comment. A qualified statistician is required.

Are all the source data underlying the results available to ensure full reproducibility?

Yes

Is the study design appropriate and is the work technically sound?

Yes

Are the conclusions drawn adequately supported by the results?

Yes

Are sufficient details of methods and analysis provided to allow replication by others?

Yes

Reviewer Expertise:

Geriatrician

I confirm that I have read this submission and believe that I have an appropriate level of expertise to confirm that it is of an acceptable scientific standard.

# Reviewer report: Reviewer response for version 1

**DOI:** 10.5256/f1000research.134559.r254035

**Published:** 2024-03-09

**Authors:** Roger E. Thomas

**Affiliations:** 1 Department of Family Medicine, Cumming School of Medicine, University of Calgary, Calgary, AB, Canada

This is an important topic. There is a limited literature on the outcomes of care provided by home visits of physicians or visiting nurses or other health practitioners. Carefully conducted RCTs at low risk of bias and appropriately powered are needed.

 The computations for phase 3 (those who consented to receive ECM) concluded 14!! respondents were needed. This computation does not take into account the need for generalizability of results and is speciously scientific.

 You stated 53% had a chronic medial issue. How many had 2, 3 .. such issues?

 You commented that 87% had regular caregivers. What did these caregivers provide? provide compared to ECM? and did the caregivers and ECM work together? What were their combined results?

 An interesting approach, but tiny sample, no RCT, and low generalizability.

Is the work clearly and accurately presented and does it cite the current literature?

Yes

If applicable, is the statistical analysis and its interpretation appropriate?

Partly

Are all the source data underlying the results available to ensure full reproducibility?

Partly

Is the study design appropriate and is the work technically sound?

Partly

Are the conclusions drawn adequately supported by the results?

Partly

Are sufficient details of methods and analysis provided to allow replication by others?

Partly

Reviewer Expertise:

Geriatric polypharmacy , hospital readmissions

I confirm that I have read this submission and believe that I have an appropriate level of expertise to confirm that it is of an acceptable scientific standard, however I have significant reservations, as outlined above.

# Reviewer report: Reviewer response for version 1

**DOI:** 10.5256/f1000research.134559.r141662

**Published:** 2022-09-01

**Authors:** Sawaeng Watcharathanakij

**Affiliations:** 1 Department of Pharmacy Practice, Faculty of Pharmaceutical Science, Ubon Ratchathani University, Ubon Ratchathani, Thailand

This is a very interesting research article. However, the abstract needs a minor change to show attributes of ECMs rather than the method used (group talks). In addition, some changes are required e.g. statistical formula, the number in Table 1 is not consistent with narrative content, additional explanation at some points, and others as highlighted in the attached file. Overall, minor revision is required. Please see the attached file for revision.

Is the work clearly and accurately presented and does it cite the current literature?

Yes

If applicable, is the statistical analysis and its interpretation appropriate?

Yes

Are all the source data underlying the results available to ensure full reproducibility?

Yes

Is the study design appropriate and is the work technically sound?

Yes

Are the conclusions drawn adequately supported by the results?

Partly

Are sufficient details of methods and analysis provided to allow replication by others?

Yes

Reviewer Expertise:

health services research, pharmacoepidemiology, drug utilization study, instrumental development, longitudinal design, and longitudinal statistical models.

I confirm that I have read this submission and believe that I have an appropriate level of expertise to confirm that it is of an acceptable scientific standard.
